# Supplementary material for: Evaluation of Clobetasol and Tacrolimus Treatments in an Imiquimod-Induced Psoriasis Rat Model
Source: Int J Mol Sci. 2024 Aug 26;25(17):9254. doi: 10.3390/ijms25179254 (PMC11395139; doi:10.3390/ijms25179254)
Supplement: Supplementary file 1 [file ijms-25-09254-s001.zip › ijms-3149965-supplementary.pdf]

Supplementary figures

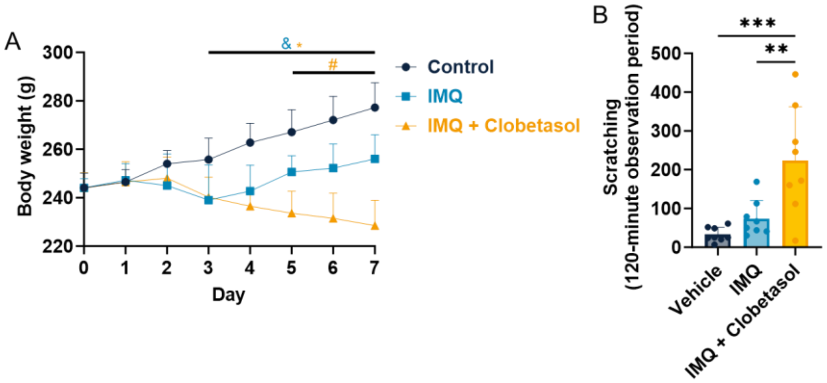

Figure S1. Effects of treatments on male rats' body weight and scratching behavior upon Imiquimod. A. Evaluation of rat body weight upon Imiquimod (IMQ, topical administration, 37.5 mg/cm²) with or without Clobetasol (topical administration, 30 mg/cm²). Treatments were administrated daily for up to 7 days. B. Evaluation of scratching behavior upon treatments. Statistical differences between the groups were determined using a mixed-effects model (REML, groups and time as factor) followed by Tukey's multiple comparisons test: & $p \leq 0.05$ control vs. IMQ, * $p \leq 0.05$ control vs. IMQ + Clobetasol, # $p \leq 0.05$ IMQ vs. IMQ + Clobetasol, ** $p \leq 0.01$, *** $p \leq 0.001$. Data represent mean and SD. n = 8 rats per group.

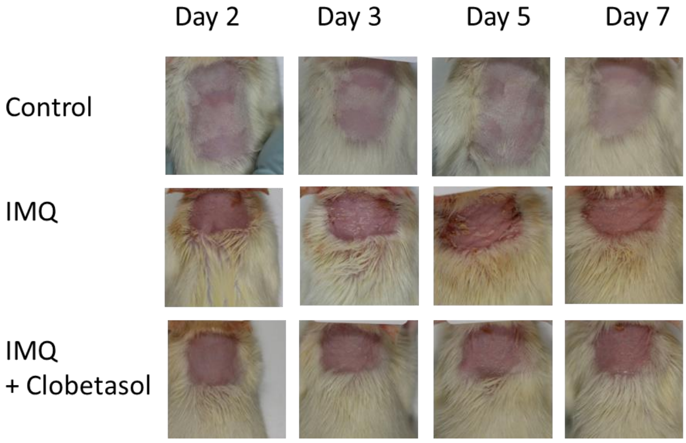

Figure S2. Evolution of dorsal skin damage over the time in control female rats (no application) and Imiquimod-treated rats (IMQ, topical administration, 37.5 mg/cm2) with or without Clobetasol (topical administration, 30 mg/cm2).

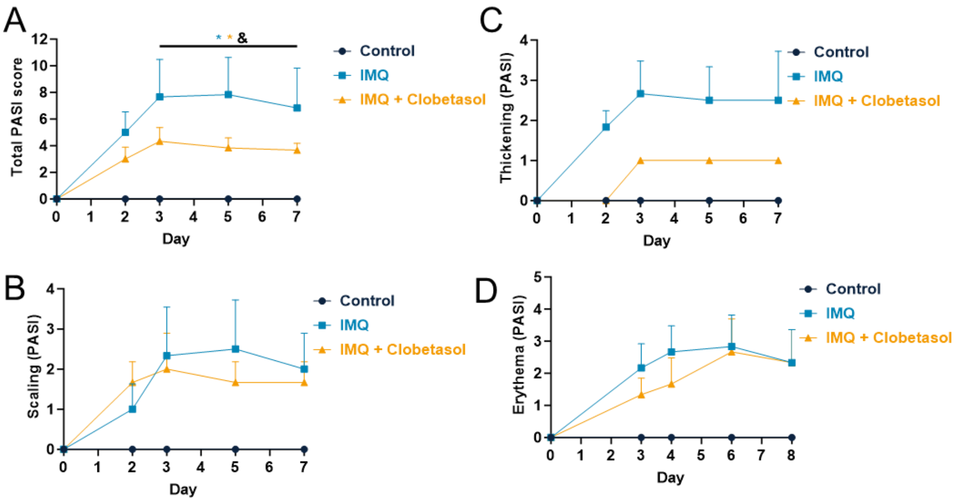

Figure S3. Effects of treatments on female rats' PASI score upon Imiquimod (2nd experiment). A. Evaluation of the cumulative total PASI score (including Thickening, Scaling, and Erythema) upon Imiquimod (IMQ, topical

administration, 37.5 mg/cm²) with or without Clobetasol (topical administration, 30 mg/cm²). Treatments were administrated daily for up to 7 days. B. Evaluation of Thickening upon treatments. C. Evaluation of Scaling upon treatments. D. Evaluation of Erythema upon treatments. Statistical differences between the groups were determined using a mixed-effects model (REML, groups and time as factor) followed by Tukey's multiple comparisons test: * p ≤ 0.05 control vs. treatments, # p ≤ 0.05 IMQ vs. IMQ + Clobetasol. Data represent mean and SD. n = 6 rats per group.

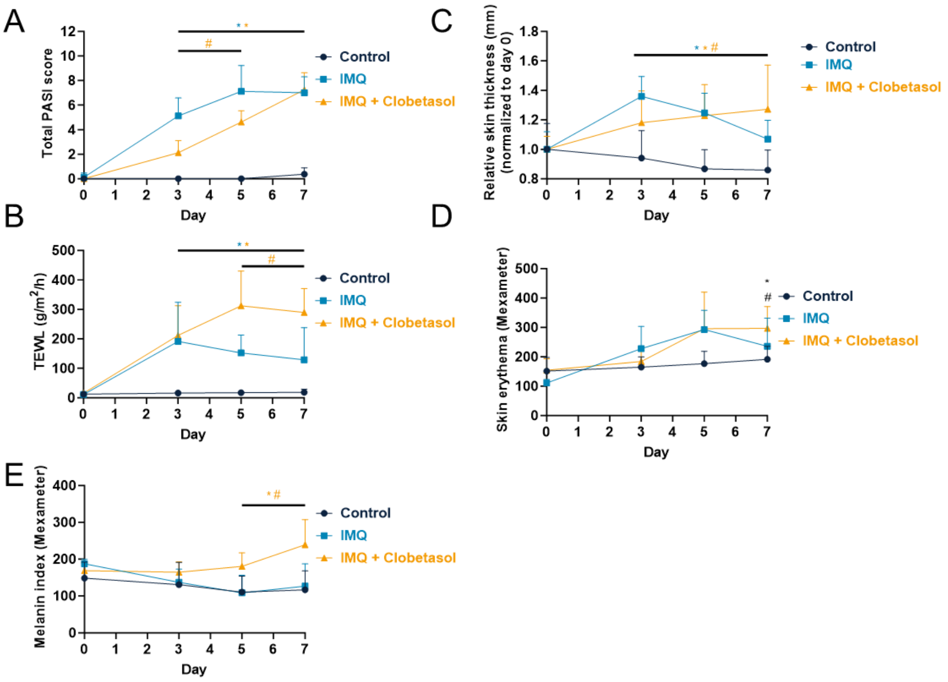

Figure S4. Effects of treatments on male rats' PASI score and skin inflammation upon Imiquimod. A. Evaluation of the cumulative total PASI score (including Thickening, scaling, and Erythema) upon Imiquimod (IMQ, topical administration, 37.5 mg/cm²) with or without Clobetasol (topical administration, 30 mg/cm²). Treatments were administrated daily for up to 7 days. B. Evaluation of skin erythema upon treatments. C. Evaluation of Transepidermal Water Loss (TEWL) upon treatments. D. Evaluation of skin erythema using a Mexameter. E. Evaluation of Melanin index using a Mexameter. Statistical differences between the groups were determined using a mixed-effects model (REML, groups and time as factor) followed by Tukey's multiple comparisons test: * p ≤ 0.05 control vs. treatments, # p ≤ 0.05 IMQ vs. IMQ + Clobetasol. Data represent mean and SD. n = 8 rats per group.

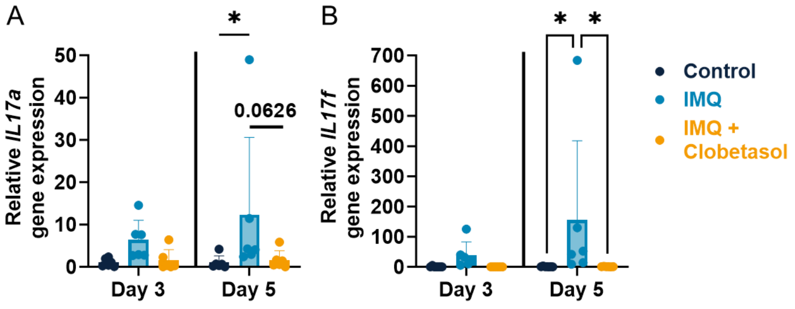

Figure S5. Effects of treatments on female rats' Interleukin 17 gene expression upon Imiquimod. A. Evaluation of the *IL17a* gene expression by RT-qPCR on day 3 and 5 upon Imiquimod (IMQ, topical administration, 37.5 mg/cm²) with or without Clobetasol (topical administration, 30 mg/cm²). Treatments were administrated daily for up to 7 days. B. Evaluation of the *IL17f* gene expression by RT-qPCR on day 3 and 5. Statistical differences between the groups were determined using a one-way ANOVA followed by Tukey's multiple comparisons test: * p ≤ 0.05. Data represent mean and SD. n = 6 rats per group.
